# Intrahousehold management and use of nutritional supplements during the hunger gap in Maradi region, Niger: a qualitative study

**DOI:** 10.1186/s40795-019-0329-0

**Published:** 2020-03-03

**Authors:** Caroline Marquer, Céline Langendorf, Lynda Christelle Woi-Messe, Fatou Berthe, Eric-Alain Ategbo, Santiago Rodas-Moya, Saskia dePee, Rebecca F. Grais

**Affiliations:** 1 Epicentre, Paris, France; 2 Unicef, Abidjan, Abidjan, Côte d’Ivoire; 3 Unicef, Niamey, Niger; 4 Nutrition Division (OSN), World Food Programme, Rome, Italy; 5 Nutrition and Health Division, Wageningen University, Wageningen, Netherlands; 6 Friedman School of Nutrition Science and Policy, Tufts University, Boston, MA USA

**Keywords:** Malnutrition, Children, Distribution, Management, Perception, Supplementary food, Niger, Maradi

## Abstract

**Background:**

Nutritional supplements are used for preventing and treating childhood malnutrition. While there is a growing body of evidence on product efficacy, less emphasis has been placed on how they are perceived and used at the household level. Here, we report on the intrahousehold management of three different supplements (Ready to Use Supplementary food (RUSF), medium quantity lipid-based nutrient supplements (LNS-MQ) and Super Cereal Plus (SC+)) in the region of Maradi (Niger). The main objective of this study was to describe the use, consumption and perception of the three different nutritional products at the household level.

**Methods:**

The study was conducted in the Madarounfa district in the region of Maradi (February – March 2012). Female caregivers were purposely selected from eligible households and invited to participate. Data were collected through focus group discussion and interviews and were analyzed using thematic content analysis.

**Results:**

In total, 114 caregivers participated. Three major themes were initially identified and included preparation and conservation; consumption and sharing practices as well as perception of impact. The data showed good acceptance at the household level including perceived benefits for the target children, health improvement, prevention of illness and malnutrition. Sharing and gifting at both household and community level were also reported.

**Conclusions:**

Caregivers displayed positive perceptions toward the investigated supplements. Patterns of actual management should be considered in the design, implementation, monitoring and evaluation of future programs.

## Background

In Niger, with only one harvest per year and an annual lean period from June to October, food insecurity is one of the leading causes of acute malnutrition and mortality in children [1–8]. To mitigate the impact, a wide range of nutritional supplements, which differ in energy content, nutrient composition and packaging, are used for preventing and treating malnutrition among children [9–14]. While there is a growing body of evidence on the individual efficacy of these products, less emphasis has been placed on how these supplements are perceived and integrated within familial eating practices [15–20].

This qualitative study was an additional component of a parent intervention study comparing the effectiveness of different strategies to prevent acute malnutrition among children aged 6–23 months: a nutritional supplement alone, a nutritional supplement and household support through a food ration or cash transfer, or a cash transfer alone [21–23]. The objective was to provide additional understanding of how nutritional supplements are managed in the home and their acceptability to guide nutritional programming in settings like Maradi aimed at reducing the incidence of severe acute malnutrition (SAM) [24]. The three supplements were a ready to use supplementary food (RUSF, Supplementary Plumpy®), a Lipid-based Nutrient Supplement Medium Quantity (LNS-MQ, Plumpy’Doz®), and improved corn-soy blend for young children also known as Super Cereal Plus (SC+).

RUSF is a Lipid-based Nutrient Supplement Large Quantity (LNS-LQ), packed in individual sachet of 100 g, recommended for 6 months old children at the dosage of one packet per day per child. LNS-MQ is packed in individual pot of 325 g gr, recommended for 6 months old children at the dosage of 3 spoons per day per child, one pot per week. SC+ is a Corn Soya Blend, for children from 6 to 24 months, packed in 1.5 kg bag. Contrary to the two other supplements described, it is consumed like porridge by mixing an appropriate proportion of flour and clean water followed by a cooking time at simmering point from 5 to 10 min and need to be consumed immediately (Table 1).

**Table 1:** Profile of nutritional supplements per daily ration

	LNS-MQ Plumpy’Doz®	RUSF Supplementary Plumpy®	Enriched flour Super Cereal Plus (SC+)
Packaging	Pot of 325 g	Sachet of 92 g	Bag of 1.5 kg
Daily ration	46 g	92 g	200 g
Instructions for use	3 spoons per day	1 sachet per day	4 porridges per day (50 g and 250 ml of water)
Quantity distributed/months	4 pots	30 sachets	4 bags
Presentation aspects	Plastic container	Individual packet	Requires water and utensils to mix and serve

*RUSF* Ready to Use Supplementary food, *LNS-MQ* Medium-quantity lipid-based nutrient supplement

Perception is defined here as how participants described the products, the impact on their children as well as beliefs and opinions about the products. Of the little research to date, ready to use foods appear well accepted in households [25, 26]. Nevertheless, there has not been a formal documentation of the utilization of SC+. Qualitative information can contribute to the design of nutrition interventions that foster the adequate use of products, and promote the development of healthy eating habits [27].

## Methods

### Design

The qualitative study was embedded in an intervention study aiming to compare the incidence of malnutrition and mortality among children aged 6 to 23 months receiving different strategies to prevent acute malnutrition [21]. The qualitative study was conducted in February 2012, 6 months after the first distribution. Two villages in each intervention arm were purposefully selected from the list of villages selected for the overall research based on their diversity in terms of population size, location, access and distance to healthcare facilities and marketplaces. One has the highest population size, was nearest from a health center, market and water access, easily accessible by road and the second had the lowest population size, farthest from a health center, market and water access, and not as easily accessible by road. Data from those villages are reported here. Available female caregivers of children enrolled in the intervention study were randomly selected from an exhaustive list of participants and invited to participate in focus group discussions (FGD) or in-depth interviews (IDI) [28]. Only one woman per household or compound was eligible. Socio-demographic data of the participants were not collected. Women were selected as they were the adult who went to collect the products at the distribution sites and were responsible for giving it to the child. Prior to receiving the monthly distribution, caregivers took part in an educational session, focusing on the use of the nutritional supplements and essential nutrition feeding actions adapted to each age group [29].

### Data collection and analysis

Focus group discussions were conducted in the participating communities with between 6 to 8 participants [30–32]. An interview guide (Additional file 1) was developed using open-ended and semi-directed questions targeting the themes related to the objectives of the study: preparation and conservation, consumption and sharing practices and perceived impact of the consumption of the nutritional supplements. All the discussions were conducted in a private area in the villages using local language (Hausa) and audio-recorded with permission from the participants. The recordings were directly transcribed in French by a local translator and then from French to English by another translator. The transcripts were verified by both F.B. and C.M.. Data focusing on supplementary food (RUSF, LNS-MQ and SC+) were analyzed manually by two researchers. Other qualitative data concerning cash transfer were analyzed separately and reported elsewhere [33]. Thematic content analysis, using both deductive and inductive approaches, was applied to code the transcripts [34–37].

Results are presented by themes and are supported with quotes as illustration of participants’ responses.

## Results

A total of 114 caregivers participated (Table 2).

**Table 2:** Discussions by intervention group, number of participants

Nutritional supplement distributed	Number of participants
RUSF/Cash	16
SC+	28
RUSF	26
LNS-MQ/Cash	16
SC+/Cash	15
SC+/Food ration	13
Total	114

When referring to RUSF and LNS-MQ, most of the participants named the nutritional supplements by a word in Hausa representing its consistency and appearance, they were primarily described as “biskit” (biscuit) or “labou” (groundnut paste).

*“Giving the children biscuits protected them against certain illnesses like diarrhea, vomiting and fever”*, RUSF/Cash.

*“We are very happy with the biskit because it protects our children from malnutrition”*, LNS-MQ/Cash.

When referring to SC+, consistency, appearance, perceived effects on weight gain and overall nutritional status were also considered. Participants described SC+ as “gari”, which means “flour”, but also as “garin kwamasso”, which means flour for malnutrition, or “garin boudous”, meaning flour that promotes weight gain, or “garin taimako”, which means aid flour.

### Preparation

During the distribution and prior to returning home, caregivers were invited to participate in an information session on how to use the nutritional supplements and answer any questions. During the interviews, some of the mothers described deviations in the use of the supplement at the household level. For example, some caregivers reported that they mixed the LNS-MQ into food or drink to prevent vomiting or with hot milk to increase appetite. Some reported that they did not mix the RUSF, as they were instructed.

*“I added water and give him like a liquid, he accepted to drink it because I noticed that when he eats this compact paste he had to vomit immediately”.* LNS-MQ.

*“I give it to my child as it is, I had never mixed it with something else”.* RUSF.

Regarding SC+ consumption, most frequently, the mode of preparation consisted of cooking it with water, as presented during the information sessions. However, some participants said that they use it “moistened” for which they covered the flour with hot water, as for preparing local couscous. Other participants indicated consuming the SC+ in “dry” form i.e. without any preparation. Some participants explained that they prepared it depending on the children’s preferences and some reported adding other ingredients such as oil, onion, peanut meal, salt, or sugar. A participant indicated:

“*That’s what I do, I buy some oil, some onion and then peanut meal and I mix it with a little salt”.* SC+.

Different forms of preparing the nutritional supplements emerged.

*Consumption and sharing practices.*

Divergences in the way in which nutritional supplements are consumed, emerged as a commonality among participants. However, although differences in how the nutritional supplements were given to children emerged, when referring to LNS-MQ and RUSF, most of the participants explained that they complied with the daily dose of one packet, most administered it between meals, in the morning. However, some participants indicated dividing the daily dose into two or three feedings, particularly for the youngest children who were still breastfeeding. A participant said:

*“When the distribution started, my child was not yet complementary feeding. When I take the packet I divide it in two, I give one portion in the morning, the rest in the evening; but now that he is weaned, he eats a packet by himself”.* RUSF.

When exploring how LNS-MQ was used, most of the participants indicated that they gave “one spoonful in the morning, one spoonful at noon, one spoonful in the afternoon.” However, some participants indicated that their children disliked the product, particularly the first few days after introduction. These participants explained that when children disliked the product, they tried to find ways to make their children like it. Hence, finding ways to increase acceptability were the main drivers to find alternative ways to “prepare” LNS-MQ.

A participant explained:

*“I mixed it* [referring to LNS-MQ] *with beans and rice for him at first when I saw that he didn’t want to eat it. [ …*] *but now he’s used to it, he eats it without me mixing it into other things”.* LNS-MQ/Cash.

Most of the participants indicated that color and consistency of prepared SC+ helped them to know if the child would consume it or not. A participant explained,

*“After preparation, we use it for up to 30 minutes. After this there is a change in color or it may become liquid as water meaning that it then it becomes less concentrated”.* SC+.

In reference to RUSF, LNS-MQ and SC+ most of the participants reported that when the supplements were introduced to the children for the first time, they were not well accepted, but once they got used to them, they appreciated the products. Referring to SC+, a participant explained:

*“In the beginning, my child didn’t want to eat it. I waited and offered him to try again and again, until he accepted it. Sometimes I buy little cakes and promise to give him after he consumes the porridge”.* SC+.

From the participant’s perspective, nutritional supplements seem to be shared primarily with siblings in the household, but also with other family members, neighbors and friends.

In some households, where men had more than one wife, it was common for them to share the products with other children and even with all members of the household. A participant explained:

*“Honestly, all of the members of the household eat it and some spouses, when you refuse to give them any, they express their displeasure [ …*] *our wish is to tell you to give us a little more or to give us something else other than gari”.* SC+.

When referring to LNS-MQ a participant explained:

*“... when they provide you with labou the others expect, hope that you will share it with them, now if you refuse then trouble starts”.* LNS-MQ/Cash.

Some women also indicated sharing the supplements with close family members who lived in the same compound and/or had many children to feed. However, although sharing was frequently reported, many participants indicated keeping most of the ration for the children for whom the nutritional supplement was indicated. When referring to SC+, a participant explained:

*“Yes of course his brothers* [of the target child] *like gari, I give them a little raw gari but not the same amount as the target child and I only do it to get them out of the way because they are big”.* SC+.

This practice was also found among participants who received LNS. A participant indicated:

*“I have other children and once I get back to the house, after I have given half a pot to my co-wife, I give half a pot to my own children; I keep the remaining three pots for the target child and that covers about two weeks”.* LNS-MQ/Cash (During the distribution, the participants received four pots of LNS-MQ per child per month).

RUSF, LNS-MQ and SC+ were shared. Most of the participants indicated that they need to take care of their children and please them equally. Feeding them with nutritional supplements, or any other food, was one way of taking care of children and to please them.

Some participants indicated:

*“We make certain gestures out of obligation without wanting to when a parent comes to your house to tell you: today my child hasn’t even had a bite to eat; and so you are obligated to give them some flour”,* SC+.

“...*you put some* [referring to SC+] *in a quart scoop and you bring it to your mother so she can cook it for your brothers”*, SC+.

*“I take two packets that I hide for my daughter and the rest I give to neighbors, in-laws and my parents”,* SC+/Cash (During the distribution, the participants received four bags of 1.5 kg per child per month).

Among some breastfeeding mothers, eating one of the three supplements was perceived to help them to produce more breast milk and was an important motivating factor for consuming the supplements themselves. A breastfeeding mother indicated:

*“I’ve noticed that it increases milk supply [ …*] *I often mix the gari with oil to eat and it gives me milk”,* SC+/Cash.

*“The stock lasts for three weeks because us mothers eat some from time to time to guarantee our milk supply”,* LNS-MQ/Cash.

Only a few participants indicated that they did not share RUSF or LNS-MQ. The perception that it would not have the expected effects on the children who needed the supplement was the main reason that prevented them from sharing the products. They also indicated that the amount of supplements distributed monthly covered the entire period between two distributions, thus the product was only consumed by the target child. A participant explained:

*“For me, in principle I tell myself that the complete contents of the packet must be eaten by the child each day, but if I share it with a child that was not targeted I know deep inside that the product will not work because it was not eaten by the intended child only, so that’s why I never share it with other children”.* RUSF/Cash.

*Perception of the impact of supplementary foods.*

Nutritional supplements were often described to be a special kind of food that improves health, intelligence, physical appearance and protects children from diseases. After using the supplements for 5 months, the benefits described from nutritional supplements included children’s weight gain, physical strength, beauty, intelligence and health maintenance.

Independently of the type of nutritional supplement, after participants gave the products to their children for a 5-month period, the children were described as, “more healthy”, “more active”, “doing well”, “smoother”, “brighter”, “more handsome”, “more plump”, and “stronger”.

*“I am happy because giving the gari means that I don’t have to shuttle back and forth between my house and the CSI* [health center] *anymore, he doesn’t have diarrhea, he doesn’t vomit, he has gained weight and he has become active”*. *SC+/ Food ration.*

The nutritional supplements were presented by some participants as a special kind of food, which can provide protection from disease. Other caregivers compared the nutritional supplements to vitamins or even to medicine. When referring to SC+ participants indicated:

*“It’s a food but it is mixed with a strong medicine that makes the child stop being malnourished”,* SC+.

*“In my opinion, it* [referring to LNS-MQ] *is an enriched product that you give to children to protect them from diseases, improve their appetite, and the child gets better quickly”.* LNS-MQ/Cash.

Furthermore, some mothers who fed their children with RUSF indicated that feeding their children with the “biskit” give them independence because children become satiated and they asked for food less often. A participant explained:

*“Really, the*” *biskit”, in addition to protecting our children from illness, has become a means of independence for the mothers, because as soon as you offer them a “biskit”, they eat it right away, they drink some water and go play, thanks to the biscuit there are no more complaints from children clinging to their mothers’ skirts”.* RUSF/Cash.

Nutritional supplements are also perceived as an additional financial support to the household. The nutritional supplements were described as a financial impact on households by guaranteeing food for their children. Participants indicated:

*“...thanks to the gari the women’s expenses have been reduced because as soon as the child is full he doesn’t demand anything else to eat”,* SC+.

The financial impact was also described in terms of decreasing expenses for medical costs because children were sick less often. A participant explained:

*“I can say that husbands are satisfied because they said that thanks to the flour, they don’t pay medical costs anymore because the children have stopped being sick all the time”.* SC+.

## Discussion

Our qualitative findings provide valuable information on how household practices and perception can vary depending on the type of nutritional supplements and how they are integrated within familial eating practices. The data show a good acceptance of the supplements at the household level. Further, participants perceived positive effects of the supplements on children’s health, intelligence and physical appearance, and potential effect on children’s nutritional status particularly toward SC+, emerged as a possible important driver for giving these products to the children. This result is important as it is a possible driver for the parents, who perceive positive effects, which may then lead them to give the supplements to the target children to improve health status, and therefore also respecting the instructions.

The acceptability and use of the supplements showed the importance of adapted educational sessions. Such information and education sessions seem to have an impact on the consumption of the product. Adhering to the messages, promotion and education sessions should be considered as a pillar to improve intake of the supplement in addition to social norms.

Concerning the description of the supplements, findings highlighted the importance of the consistency. While RUSF and LNS-MQ were often described as provided to children adequately, few participants described the adaptation of the daily dose, interpreted as encouraging consumption by the child. With respect to SC+, its consistency was perceived as more similar to customary foods, which seems to have given more flexibility for meal preparation to women.

Another topic, which would be important to consider in a distribution to improve intake by the targeted individual of the supplement, emerged as sharing of the nutritional supplement among members of the household and community. Sharing was interpreted as having potential negative impact on the expected effect on the child’s nutritional status. The three supplements appeared all as being potentially shared. These results showed that giving the nutritional supplement only to the target child when there are also other children are living in the same household and when there is not enough food available for all household members is difficult, and not a realistic assumption.

RUSF and LNS-MQ were shared primarily with other children in the household. This finding was consistent with findings from other authors who described intra-household sharing of RUSF and LNS-MQ among children [9, 26, 38]. Our results suggest that this is primarily because RUSF and LNS-MQ are distributed in small packages and because they are ready for consumption. Small packages may contribute to the perception that the target child should consume the entire dose. In this specific context, the sharing of nutritional supplements seem to be part of a deep-seated cultural practice, or social norm, of sharing, which will have to be integrated into the sensitization messages or otherwise considered operationally [39, 40]. Likewise, solidarity toward immediate and close family members, neighbors and friends also emerged as socially desirable and as an innate cultural practice, which heavily influenced sharing of nutritional supplements with important others.

On the contrary, SC+ is packed in bags and needs to be cooked. These characteristics may contribute to sharing. It is important to note that the quantity of SC+ provided also included the assumption that part of the ration may be shared. Of note, consumption of RUSF and LNS-MQ as part of a family meal was not described. This was the case though for SC+ for which both intra- and inter-household sharing was reported. This finding is also consistent with findings of other studies that reported sharing of SC+ [13, 38]. In our study, gifting SC+ to people outside of the immediate household was also reported in all groups.

Through the educational sessions the importance of not sharing supplements in order to maximize benefit for target children was emphasized, however traditional family customs and community solidarity were important factors leading to some sharing [26].

Some limitations are worth noting when interpreting these findings. Only mothers were interviewed, as they provided the supplements to the children. To better understand intrahousehold management, perceptions and practices, other family members, fathers for example, would be an important group to include. Additionally, women’s testimonies could have been biased out of fear of not receiving additional distributions if the educational information was not practiced correctly. To mitigate this fear, participants were reassured that their responses would not impact current or future distributions. It is possible also that the three-month interval between the distribution of supplements and the qualitative study could have led to problems with recall; however, we believe this to be minimal given that the distributions were notable events for beneficiary households. Furthermore, the participants were questioned regarding their perceptions and practices over a five months distribution period (August to December 2011). They could have varied during this period, as harvesting occurred in October, thus changing the eating practices of the households, especially concerning sharing practices.

Previous studies and our findings emphasize the importance of considering the sociocultural context of participants for field interventions such as distribution of nutritious foods for specific target groups [41]. Understanding of the sociocultural context related to nutrition is important to better address the needs and better design the intervention. Implementation of such programs would need to be contextualized and adapted during distribution, monitoring and evaluation. Moreover, the impact of supplements during pregnancy and breastfeeding could be also a key component to investigate. Ensuring that pregnant and breastfeeding mothers have adequate nutrition has a positive impact for the mother and infant. This is also an important aspect to consider in terms of sharing as highlighted here. Further research is needed to improve the impact of distribution of supplemental products, taking into consideration the context and also elements such as management and use at the household level.

## Conclusions

The findings showed that the nutritional supplements were perceived to have numerous positive medical, nutritional and physical impacts on the target child. Many women who participated indicated following the recommended consumption of the nutritional supplements, but their daily practices of preparation and administration varied depending on the supplement distributed. Despite sensitization sessions and household support in some intervention groups, sharing nutritional supplements with household members and gifting it to people outside the household were reported and could have reduced the actual amounts of the products consumed by the target children.

Further research using data triangulation method could explore the conditions to be met to ensure that operational strategies for the malnutrition prevention benefits the most vulnerable.

## Supplementary information

**Additional file 1:** *Interview guide * List of main and probe questions. Guideline including the themes, main questions and probes used by the interviewers during the data collection.

## Data Availability

The datasets analyzed during the current study are available from the corresponding author on request.

## Abbreviations

FGD: Focus group discussions;
IDI: In-depth interviews
LNS-MQ: Lipid-based nutrient supplements medium quantity
RUSF: Ready to use supplementary food
SAM: Severe acute malnutrition
SC+: Super cereal plus
