# Supplementary material for: Intrahousehold management and use of nutritional supplements during the hunger gap in Maradi region, Niger: a qualitative study
Source: BMC Nutr. 2020 Mar 3;6:4. doi: 10.1186/s40795-019-0329-0 (PMC7066835; doi:10.1186/s40795-019-0329-0)
Supplement: Supplementary file 1 — Additional file 1: *Interview guide * List of main and probe questions. Guideline including the themes, main questions and probes used by the interviewers during the data collection. [file 40795_2019_329_MOESM1_ESM.docx]

“Intrahousehold perception and management of nutritional supplements”

Interview guide

1/ GENERAL INTRODUCTION (Description of the supplement received during the distribution)

*Between August and December, you participated to a distribution in the region of Madarounfa, could you please describe it? What did you receive?*

Example of probe questions:

*To start, could you show me what you received? Did you know it already?* (For the interviewer: this question will help you to focus the discussion on the supplement received and avoid any confusion with another supplement); *How do you name it? From your point of view, why is this product distributed? Who is the target?*

*From now, we will focus only on the product you received.*

2/ NUTRITIONAL SUPPLEMENTS

*Now that we have discussed the product together, could you describe how you used it in your household?* (From the moment you came back from the distribution at home). *Each of you might use the product differently, we are not here to judge who does what, but to discuss together its use.*

Example of probe questions

*For example, where did you store the product when you came back from the distribution and throughout its use? Who has access to the product in the household? How is it given to the targeted child? Is it always you who gives it? Does the request it? How many times a day does this happen? Does it happen sometimes that the child asks you for it? Does your child like the product? Does it happen sometimes that other people consume it? (For the interviewer: sharing practices could be addressed by participants).*

1. Use of the supplement within the household

| Supplement | Supplementary Plumpy | CSB++ | Plumpy Doz |
| --- | --- | --- | --- |
| Main questions | *I am going to ask you to recall the situation when you give the supplement to the targeted child. Could you describe how that works? How is the supplement used in the household? When do the child receive the supplement?* | *I am going to ask you to recall the situation when you give the supplement to the targeted child. Could you describe how that works? How is the supplement in the household? When do the child receive the supplement?* | *I am going to ask you to recall the situation when you give the supplement to the targeted child. Could you describe how that works? How is the supplement in the household? When do the child receive the supplement?* |
| Additional questions | *Could you describe how that works? How is the supplement used in the household? When do the child receive the supplement? What happens before giving it to the child? (several situations possible, she moves away with the child, gives it in the yard, inside, waiting for the other children to go to school.). Are you preparing it or someone else? Do you use the bag, or do you mix it? How many times a day? Does the child finish the bag? Did you notice when the stock ends? Have you ever had difficulties?* | *Could you explain to us how you prepare the product? When the child receives the supplements? What happens before giving it to the child? (several situations possible, she moves away with the child, gives it in the yard, inside, waiting for the other children to go to school.). Are you preparing it or someone else? Do you mix it? How many times a day? How many times do you prepare it per day? and for who? Does the child finish the preparation? Did you notice when the stock ends? What does the child say when he consumes it? When does the product finished? Do you have product remaining when you go for the next monthly distribution or does the product finished? Have you ever had difficulties?* | *Could you describe how that works? How is the supplement used in the household? When do the child receive the supplement? What happens before giving it to the child? (several situations possible, she moves away with the child, gives it in the yard, inside, waiting for the other children to go to school.). Are you preparing it or someone else? How many times a day? Does the child take the dose recommended? Did you notice when the stock ends? Have you ever had difficulties?* |

1. Perception of the supplement

*In your daily use, what is the purpose of the supplement? Does it have a specific target?*

1. Pressure or support received within the household and/or the community

*In your daily experience, have you ever felt pressured from other people when using the product or during it storage? In the same way, are there people who support you in this activity? For example, people who give the product if you are absent? People who remind you to give the product. From whom? In the household (stepmother, spouse, co-wife). What are the reasons? How do you feel about it?*

1. Impact perceived of the consumption of the supplement within the household

*Since you start using the supplement, have you noticed any changes in the child and even in the household?*

*For example, have you noticed any changes in the child's health? on his appearance? on his behavior?*

*Have you noticed any changes in other people? Have you noticed any changes in the household's food management?*

1. Sharing practices

*Last year in a previous study, participants talked about sharing practices with other persons (inside or outside the household). Could you tell us about it? We are not here to judge whether this is good or not, we are here to understand what is happening during a distribution and to discuss with you about what you think. Is the product consumed by other members of the family, siblings? Who? What? For example, the dose that remains that the child has not eaten, the same amount as the target child, half of the bag, just a little occasionally. What are the reasons?*

1. Additional comments of the participants

*We have discussed a lot of topics and I thank you very much, do you have any other comments to share with us? Would you like to discuss other points that seem important to you?*
